# Next generation sequencing yields the mitochondrial genome of the critically endangered *Sarothrura ayresi* (white-winged flufftail)

**DOI:** 10.1080/23802359.2017.1318675

**Published:** 2017-04-24

**Authors:** Morne Du Plessis, Desire L. Dalton, Hanneline A. Smit-Robinson, Antoinette Kotze

**Affiliations:** a National Zoological Gardens of South Africa, Pretoria, South Africa;; b Department of Zoology, University of Venda, Thohoyandou, South Africa;; c BirdlLife South Africa, Parklands, Gauteng, South Africa;; d Applied Behavioural Ecological & Ecosystem Research Unit (ABEERU), UNISA, Florida, South Africa;; e Genetics Department, University of the Free State, Bloemfontein, South Africa

**Keywords:** White-winged flufftail, next generation sequencing, critically endangered

## Abstract

The mitochondrial genome of *Sarothrura ayresi* was sequenced using next generation sequencing technology. The size of the genome is reported as 16,767 bp and comprises 13 protein-coding genes, 2 rRNAs and 22 tRNAs. The organization of the genome is comparable to that of other bird species. A phylogenetic comparison mapped the relative relationship of *Sarothrura ayresi* with respect to other species in the order Gruiformes.

The Critically Endangered white-winged flufftail (Sarothrura ayresi, WWFT) forms part of the genus Sarothrura (IUCN 2015). The species is known from a limited number of high altitude wetlands in South Africa with records from November to March (austral summer), and more than 4000km to the north, from only three sites in Ethiopia (from July to September-boreal summer; Taylor 1994; De Smidt 2003; Sande et al. 2008). It is estimated that there are only 50 birds left in South Africa and perhaps only 250 throughout its global range (BirdLife International 2015; Taylor et al. 2005). This small and enigmatic flufftail is threatened by the loss of its wetland habitat (Davies et al. 2015). This critical endangered status of the WWFT is certainly the biggest driving force behind the ongoing research on this bird, particularly in order to potentially develop conservation strategies that could enable the survival of the species. Sequence information on the family Sarothruridae of which the WWFT is a member, is scant in the current databases and as such is inadequate for the purpose of performing extensive genomics-based high throughput analyses to unravel information on phylogenetic relationships. Therefore, we determined the complete mitogenome sequence of the WWFT in order to serve as a resource for research.

In the present study, a single specimen collected from Wakkerstroom, South Africa (27°21’25.11"S, 30°07’13.50"E) in January 2014 was selected for mitochondrial analysis. This was performed using standard extraction using the ZR Genomic DNA^TM^ – Tissue MiniPrep Kit (Zymo Research Corporation), library preparation and Illumina high-throughput sequencing (Illumina Inc., San Diego, CA). Sequences were assembled using the CLC Genomics platform (CLC Bio, Aarhus, Denmark). The mitogenome was assembled under the *de novo* option, using the standard parameters. Various kmer sizes were used during optimisation and the full mitochondrial genome for the WWFT was assembled using kmer size 31. The mitochondrial assembly constituted 8597 reads out of the 18,567,330 that remained after quality trimming and removal of contaminating adapters, using the Trimmomatic software (Bolger et al, 2014). Average genome coverage of 53X was obtained for the assembly.

The complete mitochondrial genome of the WWFT as determined in the study is 16,767 bp in length. The overall base composition is 23.47% for A, 12.17% for C, 31.55 for G and 32.78% for T. Prediction of protein-coding genes (PCGs) was performed using MITOS (Bernt et al. 2013), DOGMA (Wyman et al. 2004) as well as BLAST (Altschul et al. 1990) and curated through manual inspection. The gene content of the WWFT conforms to a typical vertebrate structure, consisting of 13 protein-coding genes (PCGs), 22 tRNAs and two rRNAs. The gene order of the WWFT also conforms to the standard gene order in birds, using *Gallus gallus* as reference (Desjardins & Morais 1990). The 12 protein-coding genes, 2 rRNAs and 14 tRNAs are encoded on the H strand and the remaining NAD6 and 8 tRNAs (trnE, trnP, trnS2, trnY, trnC, trnN, trnA and trnQ) are on the L strand. With the exception of COX1 (GTG), ND3 (ATC) and ND2 and ND5 (both ATA), the rest of the PCGs use the standard ATG start codon. The termination codons varied between TAA, TGA and TAG. The 22 tRNAs range in size from 66 to 74 bp. The 12S rRNA is 965 bp in size whilst the 16S rRNA is 1605 bp. The mitochondrial genome and its associated annotation were submitted to the NCBI under Genbank accession number KY075897. The specimen and DNA obtained from the sample are currently stored at the Biobank of the National Zoological Gardens of South Africa.

The WWFT mitogenome constitutes the first whole mitochondrial genome entry within the Sarothruridae family. In order to evaluate its relative position within the order Gruiformes a phylogenetic tree (maximum-likelihood) was constructed to place the WWFT among species representing the Rallidae, Gruidae, Heliornithidae and Otididae Families using MEGA version 6 (Tamura et al. 2013). Whilst there are more families classified within this order, these were individuals from families that had whole mitochondrial genomes available. The *Gallus gallus* mitochondrial genome was used as outgroup. The resultant phylogenetic tree, in Figure 1, indicates that the WWFT is closer associated with members of the Rallidae (*Coturnicops exquisitis*) and Heliornithidae (*Heliornis fulica*) than with members of the Gruidae (*Anthropoides paradiseus*) and Otidae (*Otis tarda*).

**Figure 1.**
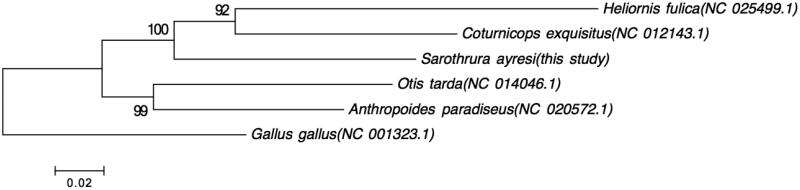
Maximum-likelihood tree based on the GTR model of five species of birds within the order Gruiformes, with bootstrap percentages indicated. The tree was rooted using *Gallus gallus* as outgroup.
